# One Thing Leads to Another: Anticipating Visual Object Identity Based on Associative-Memory Templates

**DOI:** 10.1523/JNEUROSCI.2751-19.2020

**Published:** 2020-05-13

**Authors:** Sage E.P. Boettcher, Mark G. Stokes, Anna C. Nobre, Freek van Ede

**Affiliations:** ^1^Department of Experimental Psychology, University of Oxford, Oxford OX2 6GG, United Kingdom; ^2^Oxford Centre for Human Brain Activity, Wellcome Centre for Integrative Neuroimaging, Department of Psychiatry, University of Oxford, Oxford OX3 7JX, United Kingdom

**Keywords:** alpha, attention, EEG, memory, prediction, template

## Abstract

Probabilistic associations between stimuli afford memory templates that guide perception through proactive anticipatory mechanisms. A great deal of work has examined the behavioral consequences and human electrophysiological substrates of anticipation following probabilistic memory cues that carry spatial or temporal information to guide perception. However, less is understood about the electrophysiological substrates linked to anticipating the sensory content of events based on recurring associations between successive events. Here, we demonstrate behavioral and electrophysiological signatures of using associative-memory templates to guide perception, while equating spatial and temporal anticipation (experiments 1 and 2), as well as target probability and response demands (experiment 2). By recording the electroencephalogram in the two experiments (*N* = 55; 24 females), we show that two markers in human electrophysiology implicated in spatial and temporal anticipation also contribute to the anticipation of perceptual identity, as follows: attenuation of alpha-band oscillations and the contingent negative variation (CNV). Together, our results show that memory-guided identity templates proactively impact perception and are associated with anticipatory states of attenuated alpha oscillations and the CNV. Furthermore, by isolating object–identity anticipation from spatial and temporal anticipation, our results suggest a role for alpha attenuation and the CNV in specific visual content anticipation beyond general changes in neural excitability or readiness.

**SIGNIFICANCE STATEMENT** Probabilistic associations between stimuli afford memory templates that guide perception through proactive anticipatory mechanisms. The current work isolates the behavioral benefits and electrophysiological signatures of memory-guided identity-based anticipation, while equating anticipation of space, time, motor responses, and task relevance. Our results show that anticipation of the specific identity of a forthcoming percept impacts performance and is associated with states of attenuated alpha oscillations and the contingent negative variation, extending previous work implicating these neural substrates in spatial and temporal preparatory attention. Together, this work bridges fields of attention, memory, and perception, providing new insights into the neural mechanisms that support complex attentional templates.

## Abstract (precis)

Probabilistic associations between stimuli afford memory templates that guide perception through proactive anticipatory mechanisms. A great deal of work has examined the behavioral consequences and human electrophysiological substrates of anticipation following probabilistic memory cues that carry spatial or temporal information to guide perception.

## Introduction

Probabilistic associations between stimuli can lead to memory-based templates that impact perceptual performance through anticipation of the location, temporal onset, identity, or features of anticipated sensory events. Early studies relied on relatively simple symbolic cues (e.g., arrows) to demonstrate that attention can be guided in space and time, and across features to facilitate performance (Posner, 1980; Coull and Nobre, 1998; Treue and Martínez Trujillo, 1999). More recently, studies have considered attentional orienting in more naturalistic tasks, in which the contents of long-term memory, often probabilistic in nature, guide the processing of incoming stimuli (Hutchinson and Turk-Browne, 2012). The bulk of the studies investigating memory-guided attention have focused primarily on anticipating spatial location (Chun and Jiang, 1998; Summerfield et al., 2006; Awh et al., 2012; Goldfarb et al., 2016; Jiang, 2018) and the expected temporal onset of items (Olson and Chun, 2001; Cravo et al., 2017). However, in addition, there is mounting interest in investigating mechanisms that support memory-based anticipation of the identity of upcoming percepts (Summerfield et al., 2008; Turk-Browne et al., 2008, 2010; Kok et al., 2012; Peelen and Kastner, 2014; Stokes et al., 2014).

Identity anticipation through “perceptual templates” plays a central role in theories of attention (Duncan and Humphreys, 1989; Wolfe, 1994; Desimone and Duncan, 1995). In much of the work examining perceptual templates to date observers are explicitly provided with the template of the forthcoming target. That is, they are shown a particular object that they must subsequently match or search, such as in delayed-match-to-sample or visual search tasks (Chelazzi et al., 1993, 1998; Carlisle et al., 2011; van Driel et al., 2017). Though this can be informative in assessing perceptual templates, it fails to capture a common everyday experience in building memory templates. Outside of the laboratory, frequent associations between successive different stimuli support the establishment of memory templates. Building on previous work investigating associative memory templates (Higuchi and Miyashita, 1996; Rainer et al., 1999; Turk-Browne et al., 2008, 2010; Kok et al., 2012, 2014, 2017), we here targeted two specific human electrophysiological substrates of associative memory templates during the anticipatory period.

We developed a task to investigate the anticipation of visual identity information based on probabilistic associative memory. We report robust behavioral benefits on target perception in the context of a demanding visual identification task. We also investigated the electrophysiological markers linked to proactive template-based anticipation, specifically testing for the involvement of two canonical neural markers of anticipation from the spatial and temporal orientating literatures—the modulation of alpha-band oscillations and the contingent negative variation (CNV).

Alpha attenuation has been associated with both spatial (Worden et al., 2000; Thut et al., 2006; Haegens et al., 2011; van Ede, 2018) and temporal (Rohenkohl and Nobre, 2011; Zanto et al., 2011; van Ede et al., 2017a; Heideman et al., 2018) orienting of attention, including during long-term memory-guided anticipation (Stokes et al., 2012). Likewise, the CNV is an event-related potential (ERP) component classically associated with temporal anticipation (Miniussi et al., 1999; Nobre, 2001; Los and Heslenfeld, 2005; Pfeuty et al., 2005; Praamstra et al., 2006; Cravo et al., 2011), and also in the context of long-term memory-guided anticipation (Cravo et al., 2017). Probing the involvement of these electrophysiological signatures during object–identity anticipation is important to inform a relevant and current theoretical debate about the nature of such markers. Alpha and CNV modulations during anticipation in space and time may purely reflect changes in the excitability of underlying neuronal populations (Romei et al., 2008, 2010; Benwell et al., 2017; Iemi et al., 2017; Samaha et al., 2017), independent of “informational content.” In the current work, we isolate identity anticipation and control for general “readiness” or “excitability” by equating spatial and temporal anticipation as well as target and response probabilities. If alpha and CNV modulations nevertheless still occur under these conditions, this would provide evidence that they also play a role in the anticipation of visual content.

## Materials and Methods

### 

#### Participants

In both experiments, all participants were right handed with normal/corrected-to-normal vision had no history of neurologic disorders, and were not taking any neurologic medication. All participants gave informed written consent, and were compensated £15 per hour for a total of £45. The experiments were approved by the Oxford Central University Research Ethics Committee.

In experiment 1, 30 volunteers participated. Of the 30 participants, 5 missed >80% of the difficult targets preceded by a nonpredictive stimulus 1 (S1). On this basis, these participants were excluded from the analysis. Of the 25 remaining participants, the average age was 24.2 years (age range, 18–33 years) and there were 9 females.

In experiment 2, 36 volunteers participated. Of the 36 participants, 6 performed at chance for targets on nonpredictive S1 trials. On this basis, these participants were excluded from the analysis. Of the remaining 30 participants, the average age was 27.1 years (age range, 20–34 years) and 15 were females.

#### Procedures

Participants sat in a dimly lit booth at a distance of 100 cm from the monitor (22 inch SyncMaster 2233, Samsung; resolution, 1680 × 1050 pixels; refresh rate, 100 Hz; screen width, 47 cm). The experimental script was generated using Psychophysics Toolbox (Brainard, 1997) on MATLAB (version 2014b, MathWorks). Participants were instructed to refrain from excessive blinking and to keep their face as relaxed as possible to avoid muscular artifacts in the EEG recordings.

##### Experiment 1.

**Figure 1.**
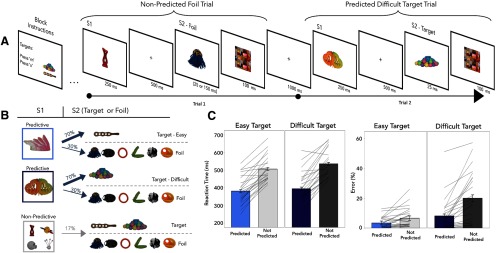
Trial schematic and behavioral data from experiment 1. ***A***, An example of the trial sequence from experiment 1. On each trial, participants saw S1, which could either be predictive or nonpredictive about the following S2, which could be an easy target (150 ms), a difficult target (25 ms), or a foil (25 or 150 ms). S2 was immediately followed by a mask. Participants were instructed to respond to the targets (but not the foils) with the corresponding button as quickly as possible. ***B***, The probability of a specific S2 target following a predictive S1 was 70%, whereas nonpredictive S1s were equally likely to be followed by either of the two potential targets or any of the four foils. S1–S2 relationships were made explicit to participants before starting the experiment. ***C***, Participants responded more quickly and more accurately to targets preceded by a predictive S1 as well as to easy targets. Additionally, there was a significant interaction in both RT and percentage error, indicating that predictive S1s had a larger benefit in the difficult-target trials.

The structure of experiment 1 is shown in Figure 1. Participants were shown a random sequence of objects taken from a set of 14 objects from the Novel Object and Unusual Name database (NOUN; Horst and Hout, 2016). Among these objects, there were four critical objects: easy S1, easy target, difficult S1, and difficult target, and 10 neutral objects. These four objects were randomly allocated to every fourth participant and then counterbalanced for subsequent participants such that for each random allocation of four objects, each object held each of the four critical roles. Participants' task was to press a corresponding key (either “m” or “x” key) whenever they detected a target. The targets switched their association with the keys randomly between blocks, such that each target was associated with the “x” and “m” buttons for half of the blocks.

Before the start of the task, observers were informed about the S1 objects. Specifically, they were told that following the presentation of a predictive S1 there was a 70% probability that the next item would be the corresponding target (i.e., the paired associate). Therefore, within the stream, specific S1 identities would predict specific target identities. In the other 30% of the trials, each of the other items was equiprobable.

A single trial consisted of the following sequence: S1, blank, stimulus 2 (S2), and a mask. S1 could be either predictive or nonpredictive and was always presented for 250 ms. S2 could be either one of the targets or a foil object. S2 was immediately followed by a 100 ms mask that consisted of patches drawn randomly from the potential target items. For each set of objects, three of these masks were created and used randomly throughout the experiment. Target difficulty was determined by its exposure duration. The easy target was always presented for 150 ms before the mask, whereas the difficult target was presented for only 25 ms before the mask. The neutral objects were shown for either 150 or 25 ms equiprobably (i.e., any particular neutral object would be shown for 150 and 25 ms half of the time). The mask was followed by a 1000 ms blank before the next trial began. With this design, the appearance of S2 was completely predictable in space and time. Participants completed 14 blocks of 100 trials in total.

##### Experiment 2.

For the structure of experiment 2, see Figure 4. The stimuli, experimental setup, and EEG procedures were the same as in experiment 1. A trial was similar to that of experiment 1, with a few critical changes. On each trial, participants first saw S1 (250 ms), which again could be a predictive or a nonpredictive S1 with equal probability. This was followed by a 750 ms blank and the quick presentation of one of three targets (30 ms)—we will refer to these targets as target A, B, or C. That is, there was a task-relevant item presented on every trial. Critically, two of these items (target A and target B) were predictable based on S1, whereas the other item (target C) was always equally probable after all S1 stimuli. Following the presentation of the target and a mask (100 ms), all three potential targets appeared on the screen, and observers used the left, down, and right arrow keys to indicate which object they had just seen. The position of the three targets was randomized across trials such that observers could not prepare their response before the response screen. The stimuli were randomly allocated to each participant. With these changes to the design, every trial and item was task relevant, and participants could not prepare a specific response during the period after S1. Here, therefore, predictive and nonpredictive S1s differed only with regard to its ability versus inability to form a specific target template in anticipation of S2.

The relationships between the S1 and target items were explicitly detailed to the participants before the experiment. In total, there were eight potential S1 items. Four of these items were predictive, and four were nonpredictive. Of the four predictive S1 items, two predicted target A and two predicted target B. That is, if one of these predictive S1 objects appeared, the associated target would follow in two-thirds of the trials. In the remaining one-third of the trials, target C would appear. On nonpredictive S1 trials, all targets were equally likely. As such, throughout the experiment, all three targets were equally likely to appear such that there was no higher probability of a predictable target.

#### Behavioral analysis (experiments 1 and 2)

Behavioral data were analyzed using R (R Core Team, 2018). Reaction times (RTs) and error rates were submitted to an ANOVA implemented in the ez package (Lawrence, 2013), and *t* tests were implemented in lsr (Navarro, 2018). Effect size estimates (η_G_^2^ and d) are provided for all effects. Plotting was completed using the ggplot2 package in R (Wickham, 2009).

#### EEG acquisition (experiments 1 and 2)

We acquired EEG using Synamps amplifiers and Neuroscan data acquisition software (Compumedics). Sixty-one electrodes were distributed across the scalp using the international 10–10 positioning system. The left mastoid was used as the active reference, and we included a right mastoid measurement to derive an average mastoid reference offline. The ground was placed on the left upper arm. Additionally, vertical and horizontal electro-oculography (EOG) electrodes were used to monitor for eye blinks and eye movements. During acquisition, data were low-pass filtered by an antialiasing filter (250 Hz cutoff), digitized at 1000 Hz, and stored for offline analysis.

#### EEG preprocessing (experiments 1 and 2)

The preprocessing and analysis scripts for both experiments can be found as html files and as reproducible scripts (Jupyter notebooks; Kluyver et al., 2016) at https://github.com/SageBoettcher/identityTemplates. The preprocessing pipeline is modified from the analysis pipeline used by Draschkow et al. (2018). All EEG data analysis was conducted in MNE-Python (Gramfort et al., 2013). The data were downsampled to 200 Hz and high-pass filtered at 0.1 Hz. To regress out eye movement activity, an independent component analysis (Jung et al., 2000) was used to decompose the data, which was high-pass filtered at 1 Hz, into 60 temporally independent components. Eye movement components were detected by first correlating the filtered data with the EOG and subsequently, when needed, manually selecting a subset of typical component maps and identifying the best group match to them (Viola et al., 2009). Selected components were then removed from the data. Trials were segmented from −200 to +750 ms (experiment 1) or +1000 ms (experiment 2) relative to the onset of S1. Average activity over the 200 ms preceding the stimulus onset was used as a baseline against which all amplitudes were calculated. Finally, epochs with especially high variance were discarded. These epochs were detected through a generalized extreme studentized deviate test for outliers with an α value of 0.05 and were discarded from the analysis. On average, 34 of 1400 trials were discarded in this manner.

#### EEG data analysis (experiments 1 and 2)

##### Alpha.

For the time–frequency analysis, we used epochs from −200 to 1000 ms. Morlet wavelets were convolved with the data between 3 and 40 Hz. For each frequency, we used a fixed 400 ms time window such that the number of cycles changed with the frequency. After the time frequency transformation, activity was averaged over all posterior electrodes (P7, P5, P3, P1, Pz, P2, P4, P6, P8, PO7, PO3, POz, PO4, PO8, O1, Oz, O_2_) and contrasted between predictive and nonpredictive trials (separately for the easy and difficult conditions in experiment 1). We expressed this as a normalized difference [(predictive minus nonpredictive)/(predictive plus nonpredictive) * 100].

##### ERPs.

The ERPs were calculated by averaging trials within a participant and then subsequently averaging these waveforms across participants separately for each condition. The ERPs were averaged across a predefined set of central–posterior electrodes (P1, Pz, P2, CPz, POz) as well as central–frontal electrodes (F1, Fz, F2, AFz, FCz). These electrodes were chosen based on previous work showing peak amplitude for the CNV at electrode Fz and peak amplitude for potentials linked to retrieval at electrode Pz. We focused our analyses on these electrodes and included the immediately surrounding electrodes to increase potential sensitivity.

#### EEG statistical analysis

Inferential claims about differences between conditions were based on a cluster-based permutation test (Maris and Oostenveld, 2007) and were reported according to recommendations by Sassenhagen and Draschkow (2019).

## Results

### Experiment 1: target templates and target difficulty

In experiment 1, we investigated whether identity templates from associative memory impact perception, as well as the neural markers that may be involved in this template-based anticipation. To evaluate the adaptive utility of the identity template, we additionally asked to what extent these hypothesized effects depend on the anticipated perceptual difficulty of the target.

The structure of the experiment is shown in Figure 1. On each trial, participants saw two sequential objects (S1 and S2) followed by a mask. Whenever participants saw one of their two potential targets—always in the S2 position—they responded with a corresponding button press on a keyboard (m or x, counterbalanced across blocks). The S1 item could either be predictive or nonpredictive of the identity of the upcoming item. Predictive S1s were followed by their respective S2 target in 70% of trials. Spatial and temporal predictions were fixed, with presentation always appearing in the center of the screen after 750 ms; therefore, predictive and nonpredictive S1s differed in that only predictive S1s enabled participants to anticipate the identity of the upcoming S2 stimuli.

#### Behavioral results

To assess whether predictive S1s impact performance and whether this effect was modulated by the expected target difficulty, we conducted repeated-measures ANOVAs on RTs and error rates with S1 type (predictive and nonpredictive) and target difficulty (easy and difficult) as factors. Behavioral results are depicted in Figure 1*C*. Target difficulty and S1 type interacted significantly in both RTs (*F*_(1,24)_ = 5.4, *p* = 0.03, η_G_^2^ = 0.002) and error rates (*F*_(1,24)_ = 12.0, *p* = 0.002, η_G_^2^ = 0.08). Moreover, we found the main effects of S1 type and target difficulty for both RTs (S1 effect: *F*_(1,24)_ = 87.3, *p* < 0.001, η_G_^2^ = 0.41; difficulty effect: *F*_(1,24)_ = 7.9, *p* = 0.009, η_G_^2^ = 0.01) and error rates (S1 effect: *F*_(1,24)_ = 29.5, *p* < 0.001, η_G_^2^ = 0.21; difficulty effect: *F*_(1,24)_ = 15.4, *p* < 0.001, η_G_^2^ = 0.28). Paired-samples *t* tests (Bonferroni-corrected *p* values) revealed a significant RT benefit (i.e., faster RTs) of the predictive S1 for both easy and difficult targets (easy: *t*_(24)_ = 9.17, *p* < 0.001, *d* = 1.83; difficult: *t*_(24)_ = 9.11, *p* < 0.001, *d* = 1.82), and that the benefit of the predictive S1 was larger for difficult targets (*t*_(24)_ = 2.33, *p* = 0.03, d = 0.47). The same pattern occurred for error rates, with a significant benefit (i.e., lower errors) following versus nonpredictive S1 items in trials with an easy target (*t*_(24)_ = 2.9, *p* = 0.01, *d* = 0.59) as well as trials with a difficult target (*t*_(24)_ = 4.93, *p* < 0.001, *d* = 0.99). Once again, this benefit of predictive S1s was larger for difficult targets (*t*_(24)_ = 3.46, *p* = 0.002, *d* = 0.69). Thus, predictive objects impact performance on the target, and this benefit was particularly pronounced when the targets were difficult to perceive.

The above results considered only target-present trials. For completeness, we also analyzed foil trials to determine whether predictive S1s also led to more false alarms. We found that observers were indeed more likely to false alarm to a foil following a predictive compared with a nonpredictive S1 (*t*_(24)_ = 3.14, *p* = 0.004, *d* = 0.62; 14.5% vs 1.5% false alarms). Because the probability that a target would appear after an informative S1 was higher than the probability that a nontarget would appear (in experiment 1, but not experiment 2, as we return to it later), this increase in false alarms following predictive S1s may simply reflect a strategic decision of participants to report the target when unsure.

#### EEG results

##### Alpha

**Figure 2.**
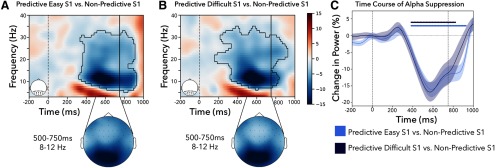
Alpha attenuation following predictive versus nonpredictive S1s in experiment 1. ***A***, Time–frequency results for posterior electrodes shows alpha attenuation in the predictive-easy S1 versus the nonpredictive trials, as well as in the predictive-difficult S1 versus the nonpredictive S1 trials. ***B***, The topographies are plotted on the same scale as the above time–frequency plot. ***C***, The time course of the alpha attenuation averaged between 8 and 12 Hz. Vertical lines at 750 ms show the onset of the S2 target. Significant clusters with a *p* value < 0.05 are denoted with the black outline (***A***, ***B***) and as horizontal lines in ***C***. Shaded areas represent ±1 SEM (68% confidence intervals).

To assess the effect of predictive versus nonpredictive S1s on induced brain activity, we first compared time-resolved and frequency-resolved maps of power (collapsed over all posterior electrodes; Fig. 2*A*,*B*, insets) from the onset of S1 until 250 ms after the onset of the S2, as seen in Figure 2. More specifically, we directly contrasted trials with a predictive and a nonpredictive S1. We did so separately for trials with a predictive S1 that predicted an easy target (predictive-easy S1) and trials with a predictive S1 that predicted a difficult target (predictive-difficult S1). The same nonpredictive S1 trials were used for both comparisons. Significant clusters emerged following both the predictive-easy S1 (Fig. 2*A*; *p* < 0.001) and the predictive-difficult S1 (Fig. 2*B*; *p* < 0.001) in comparison with following the nonpredictive S1. The maximal attenuation within these clusters for both the easy and difficult S1 occurred at ∼11 Hz and 600 ms after S1 onset (i.e., mostly concentrated within the alpha band). A topographic inspection confirmed that these effects had a clear posterior topography in line with a visual preparation effect. There were no significant clusters when directly contrasting easy to difficult S1s (all cluster *p* values > 0.13).

To have a clearer understanding of the time course of the alpha attenuation, we also averaged these effects along the classical alpha band (8–12 Hz; Fig. 2*C*). Once again, we found a significant cluster for both the easy S1s (*p* < 0.001) and the difficult S1s (*p* < 0.001), with no significant difference according to the difficulty levels during the anticipation period (*p* = 0.14, with the only cluster forming after the onset of the target).

##### ERPs

**Figure 3.**
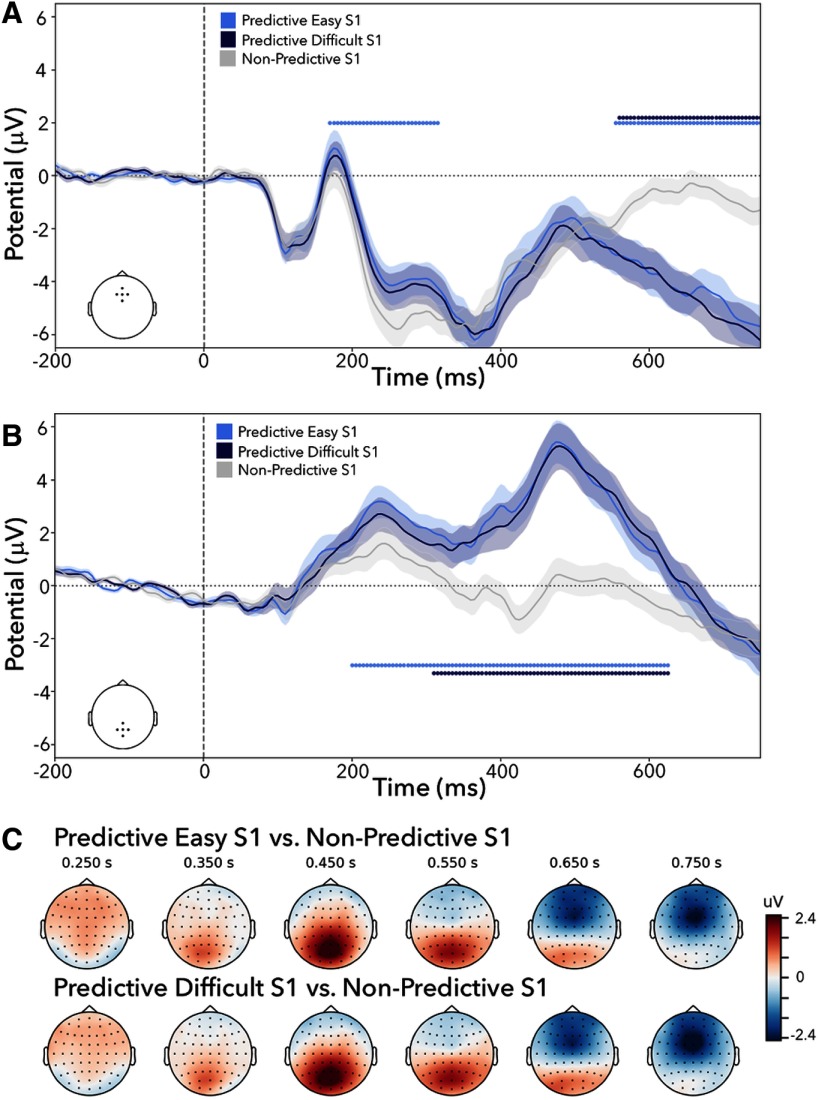
Posterior positivity and frontal negativity following versus nonpredictive S1s in experiment 1. ***A***, ERPs locked to the onset of S1 and averaged across a subset of frontal electrodes (F1, Fz, F2, AFz, FCz). Predictive S1s show a late frontal negativity relative to nonpredictive S1s, while difficulty did not significantly modulate this effect. ***B***, ERPs locked to the onset of S1 and averaged across a subset of posterior electrodes (P1, Pz, P2, CPz, POz). The predictive S1s show a clear positive deflection from the nonpredictive S1, while difficulty did not significantly modulate this effect. ***C***, Topographies of the ERP effects (predictive-easy versus nonpredictive and predictive-difficult versus non predictive) over time show an early posterior positivity followed by a late frontal negativity. Significant clusters with a *p* value < 0.05 are denoted with horizontal lines in ***A*** and ***B***. Shaded areas represent ±1 SEM (68% confidence intervals).

To investigate the anticipatory nature of identity-based templates, we additionally investigated ERPs locked to the onset of predictive-easy S1s, predictive-difficult S1s, and nonpredictive S1s for predefined clusters of frontal and posterior electrodes. The results are depicted in Figure 3. We were specifically interested in testing whether these identity-based predictions also produce a CNV—a frontal negativity—in the predefined frontal electrodes.

We first considered the frontal electrode cluster (Fig. 3*A*). For both the predictive-easy S1 and the predictive-difficult S1 cues, we found a significantly larger negativity in the late S1-S2 cue–target interval, compared with the nonpredictive S1 cues (easy, *p* < 0.001; difficult, *p* < 0.001). These negativities were associated with a frontal topography characteristic of the CNV (Fig. 3*C*). In the predictive-easy S1 condition, we additionally found an early positivity (*p* = 0.004) that is likely a spillover effect from an earlier more posterior positivity that we return to below (as also confirmed by the time-resolved topographical analysis presented in Fig. 3*C*). There were no significant clusters when contrasting the easy and difficult S1s (*p* values > 0.43).

When comparing effects for predictive versus nonpredictive S1 cues in the predefined posterior electrodes (Fig. 3*B*), a significant cluster was identified from ∼200 to 600 ms for both easy and difficult (*p* values < 0.01). The effect reflected a late positive potential elicited by predictive cues. Topographical analysis confirmed that the potential was centrally distributed over the posterior scalp (Fig. 3*C*). As with the alpha modulations and the CNV, there were no significant clusters when comparing the easy- and difficult-predictive S1s (all cluster *p* values > 0.43).

These effects were confirmed, and also nicely demonstrated, by the time-resolved topographies of predictive versus nonpredictive S1 (separated by the easy and difficult conditions), as depicted in Figure 3*C*.

### Experiment 2: target templates while equating target and response probabilities

In experiment 1, the pattern of behavioral data was suggestive of proactive and flexible template utilization, resulting in larger performance benefits when target discrimination was difficult. Proactive memory-based expectation was also suggested by alpha attenuation and a CNV following predictive versus nonpredictive S1 objects. These predictive S1s allowed participants to prepare for the identity of the upcoming stimulus, while controlling for spatial and temporal expectations that were matched between the S1 objects.

**Figure 4.**
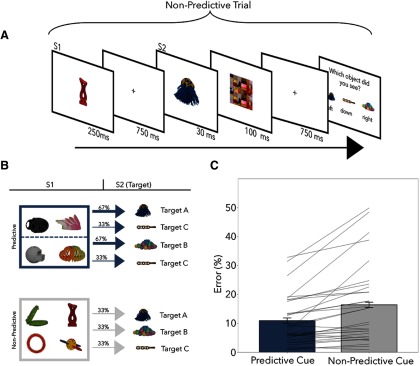
Trial schematic and behavioral data from experiment 2. ***A***, Schematic of an example nonpredictive trial in experiment 2. Participants' task was to always report the second S2 object. The paradigm is very similar to experiment 1 with the exception that participants must respond on every trial (i.e., each S2 is a target). ***B***, Probabilities of each S2 target given the preceding S1. In ***C***, we see that there is a significant effect of the predictive S1 on error rates. Because this task was a delayed forced choice, reaction times were no longer informative.

Although neural markers clearly signaled target anticipation, it was not possible to conclude that the neural effects were specifically related to the perceptual identity of the anticipated target. On average, task-relevant items (targets) were also more likely following predictive versus nonpredictive S1s, which may have led to differential motor anticipation, or states of attention. Because responses were only required to the target stimuli, during predictive S1 trials observers could not only prepare for a task-relevant visual target, but possibly also for the associated motor response. The neural effects may thus reflect general task readiness (or excitability), rather than template-specific anticipation of visual identity. To rule out this potential interpretation, we designed experiment 2 (Fig. 4).

In experiment 2, we equated these other forms of anticipation by making S2 a task-relevant stimulus on every trial. Specifically, participants were always tasked with discriminating S2, but only a subset of S1 stimuli predicted the identity of S2. Therefore, the only difference between predictive and nonpredictive S1s was the likelihood of a specific target appearing. As such, differences between the S1 conditions must be attributed to proactive target template activation. Participants once again saw predictive and nonpredictive S1s (Fig. 4*B*), which were equated for their spatial and temporal predictions, as well as motor affordances. Three stimuli served as S2, two of which were predicted by a subset of S1 stimuli and one of which was completely unpredictable. Participants responded to S2 in a three-alternative forced choice (3AFC) design. To eliminate anticipation of specific motor responses, response mappings were random on every trial. Across the experiment, all three targets were equally probable and potential differences in the preparatory period can no longer be attributed to differences in target probability or response preparation. In experiment 2, all trials had the same difficulty level, allowing us to focus exclusively on the central question of identity anticipation.

#### Behavioral results

To test for a benefit to the predictive S1s in the error rates, we used a paired samples *t* test. As seen in Figure 4*C*, targets preceded by a predictive S1 were again detected more accurately (*t*_(29)_ = 4.16, *p* < 0.001, *d* = 0.76). Because participants gave a 3AFC response after an imposed delay, reaction times were not considered informative of perceptual processing in experiment 2 and were therefore not analyzed.

#### EEG results

##### Alpha

**Figure 5.**
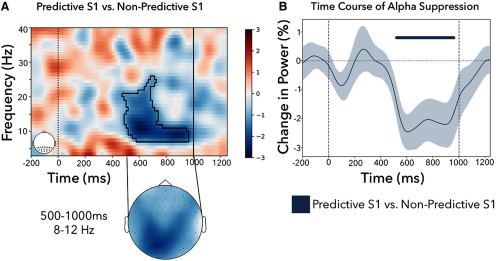
Alpha attenuation following predictive versus nonpredictive S1 in experiment 2. ***A***, Time–frequency results for posterior electrodes shows alpha attenuation following the predictive S1 relative to the nonpredictive S1, with a peak negativity at 610 ms after S1 at 11 Hz. ***B***, Time course of the alpha attenuation, averaged between 8 and 12 Hz. Vertical line at 1000 ms shows the onset of the target. Significant clusters with a *p* value < 0.05 are denoted with the black outline in ***A***, and by the horizontal line in ***B***. Shaded area represents ±1 SEM (68% confidence intervals).

To assess the alpha attenuation following predictive versus nonpredictive S1s, we compared the time–frequency maps in the period between the onset of S1 and the onset of S2. As shown in Figure 5*A*, we observed a significant cluster (*p* = 0.005), with a qualitatively similar profile (in terms of time range, frequency range, sign, and topography), as in experiment 1. The peak attenuation in this cluster was found at 11 Hz and 610 ms after S1. As in experiment 1, this attenuation was associated with a predominantly posterior topography (Fig. 5*A*). When focusing on the predefined 8–12 Hz alpha band (Fig. 5*B*), we found a significant cluster (*p* = 0.01), which spanned a similar time range as in experiment 1.

##### ERPs

**Figure 6.**
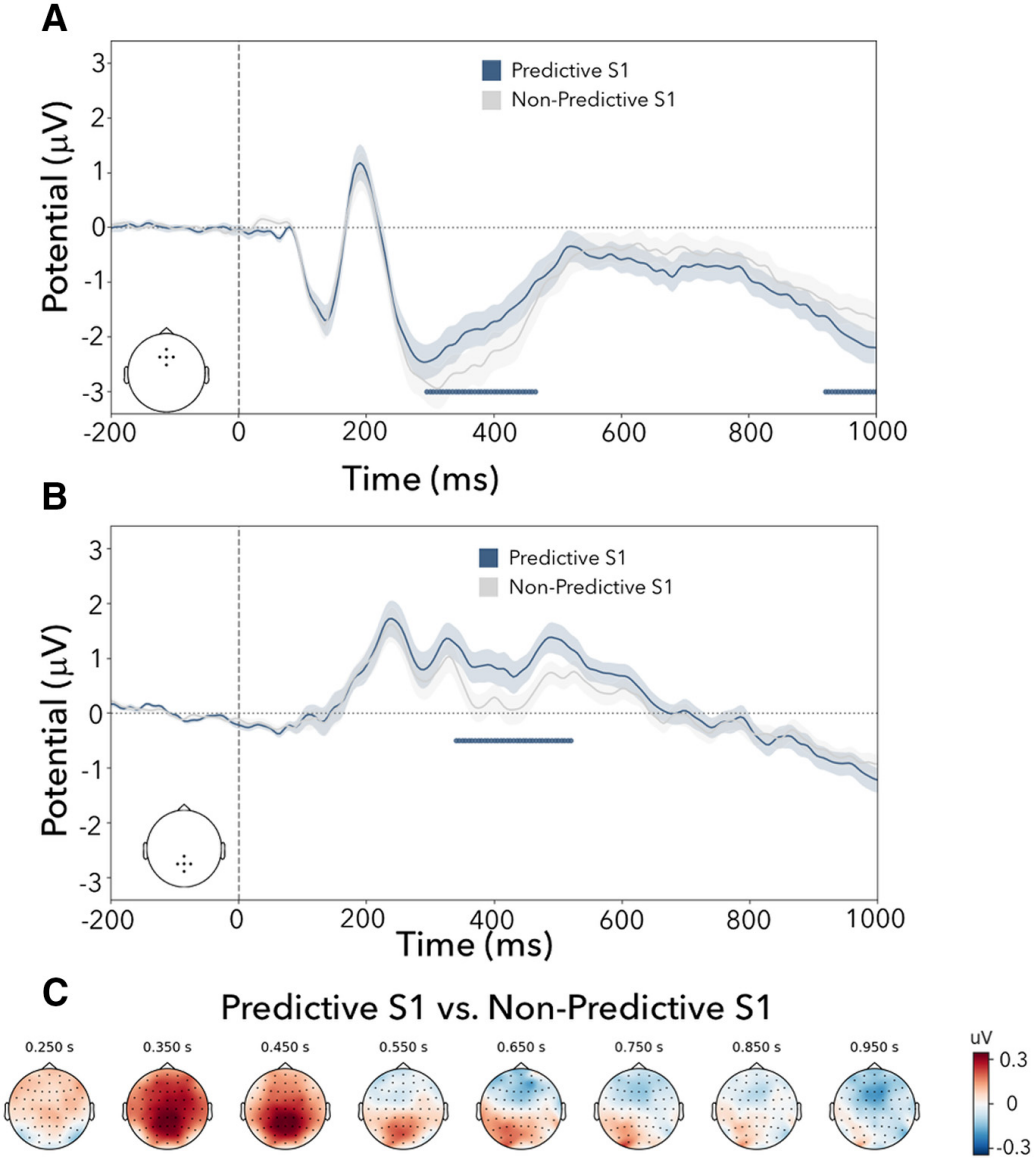
Posterior positivity and frontal negativity following predictive versus nonpredictive S1 in experiment 2. ***A***, ERPs locked to the onset of S1 and averaged across a subset of frontal electrodes. Predictive S1s show a late frontal negativity relative to nonpredictive S1s. ***B***, ERPs locked to the onset of S1 and averaged across a subset of posterior electrodes. The predictive S1s show a clear positive deflection from the nonpredictive S1s. ***C***, Topographies of the ERP effects (Predictive versus Nonpredictive) show an early posterior positivity followed by a late frontal negativity. Significant clusters with a *p* value < 0.05 are denoted with horizontal lines in ***A*** and ***B***. Shaded areas represent ±1 SEM (68% confidence intervals).

As in experiment 1, we also investigated ERPs locked to the onset of S1 in the predefined frontal and posterior electrode clusters (Fig. 6). In the frontal electrode cluster (Fig. 6*A*), we again observed a CNV—a larger negativity following predictive S1s just before the onset of S2 (cluster *p* = 0.04). Like in experiment 1, we also found a significant positive cluster in the frontal electrodes between ∼300 and 450 ms (*p* = 0.01), which again likely involved a spillover from a more posterior effect (Fig. 6*C*). Indeed, in the posterior cluster (Fig. 6*B*), predictive S1s again elicited a larger positive potential from ∼300 ms to ∼550 ms, yielding a significant cluster (*p* = 0.001).

The topographies again demonstrate how the effects of the predictive versus nonpredictive S1s develop over time and space (Fig. 6*C*), and revealed a qualitatively similar spatial–temporal progression, as observed in experiment 1.

**Figure 7.**
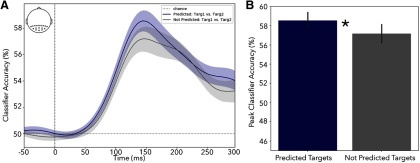
***A***, Linear discriminant analysis (LDA) classification accuracy of S2 target A versus S2 target B (in experiment 2) when preceded either by a predictive (blue line) or nonpredictive (gray line) S1. ***B***, Classifier accuracy at the peak classification time for the group average (145 ms) for both predicted and not predicted targets. To avoid circularity, the peak time was found based on the average of the predicted and not predicted data. LDA was performed in a time-resolved fashion on the baseline-corrected time series, using the topographical distribution across all posterior electrodes (as indicated in the inset) as the multivariate data features. Asterisk indicates a significant difference with a *p*-value < .05.

The tightly controlled identity–expectation manipulation in experiment 2 also enabled us to investigate whether the proactive deployment of probabilistic associative memory templates based on S1 improved neural processing of S2 during perceptual analysis (i.e., after S2 target onset). Unlike in experiment 1, the S1 items were all followed by target items, thus equating motor demands and degree of preparation. Presentation duration of S2 was also equated. To test for qualitative changes in sensory processing, we applied linear discriminant analysis to decode the content of the two predictable targets in posterior electrodes when they were preceded either by a predictive or a nonpredictive S1 (Fig. 7). Cluster-based permutations that considered the first 300 ms of target processing showed a single cluster of better decoding for predictable compared with unpredictable targets, though this did not survive cluster correction (*p* = 0.09). When we considered only the peak decoding period of all targets (at 145 ms; Fig. 7*B*), we found better decoding for predicted versus unpredicted targets (*t*_(29)_ = 2.89, *p* = 0.007). However, because this effect was not particularly strong (Fig. 7), we would like to present this as a tentative result in the hope that it will motivate further investigation, without further elaboration in the Discussion.

## Discussion

Our results provide evidence that identity templates based on probabilistic associative memory impact perception. Furthermore, these templates are associated with proactive states of attenuated alpha oscillations and the CNV, even when controlling for differences in spatial and temporal anticipation as well as response and target probabilities.

Our behavioral and EEG results build on and extend earlier work on memory-guided attentional orienting and perceptual identity templates in several ways. When considering memory-guided anticipation, we have focused here on perceptual consequences and the electrophysiological signatures of memory-guided predictions based on identity, as opposed to anticipation in space and time (Chun and Jiang, 1998; Olson and Chun, 2001; Summerfield et al., 2006; Awh et al., 2012; Goldfarb et al., 2016; Cravo et al., 2017; Jiang, 2018). We have studied this in a context where the templates must be retrieved from complex probabilistic associations in memory templates (Higuchi and Miyashita, 1996; Rainer et al., 1999; Turk-Browne et al., 2008, 2010; Stokes et al., 2009; Kok et al., 2012, 2014, 2017)—rather than being explicitly provided (Chelazzi et al., 1993; Carlisle et al., 2011; van Driel et al., 2017)—and have focused specifically on the anticipatory electrophysiological substrates associated with such templates.

This work also expands on prior work that has used paired-associate tasks similar to the one used here (Gallistel, 1990; Higuchi and Miyashita, 1996; Rose et al., 2001; Stokes et al., 2014; Brincat and Miller, 2015), but where the focus was on learning. In the current study, the focus was not on the learning of the S1–S2 associations, but rather on the exploitation of previously learned information in the service of guiding ensuing behavior (but see Rainer et al., 1999; Stokes et al., 2013, 2014) here in a demanding perceptual task with masked visual targets. Doing so, we report that participants are able to use learned identity associations to impact perception.

A major empirical contribution of our study was to identify electrophysiological markers for the anticipation of identity-related informational content in the human brain that we discuss next in turn.

### Alpha attenuation

In previous work, alpha attenuation has been noted during anticipatory periods for both spatially and temporally predictable targets (Worden et al., 2000; Sauseng et al., 2005; Thut et al., 2006; Siegel et al., 2008; Rohenkohl and Nobre, 2011; van Ede et al., 2011; Zanto et al., 2011; Heideman et al., 2018). In this context, alpha attenuation has been theorized to reflect engagement of sensory-processing areas in preparation for a task-relevant event, in line also with the notion that alpha is inversely related to firing rates (Haegens et al., 2011) and/or processing capacity (Hanslmayr et al., 2016) of the underlying populations. In our results, we have shown alpha attenuation when S1 specifically predicts the identity of an upcoming target over and above its location and temporal onset. Accordingly, we propose that the alpha attenuation also reflects engagement with visual processing areas to prepare a specific target template. As such, the alpha modulations reported here complement recent work showing that lower alpha power is associated with higher fidelity of stimulus-specific information (van Ede et al., 2018; Griffiths et al., 2019; Barne et al., 2020). In this light, it is interesting to note that alpha band oscillations were not significantly modulated by the anticipated perceptual difficulty in identifying the target, as might be expected from a pure excitability account (Romei et al., 2008, 2010; Benwell et al., 2017; Iemi et al., 2017; Samaha et al., 2017). Rather, at least in our task, the observed alpha attenuation appears to reflect the anticipation of specific visual content related to target identity, though we note that visual content in our task entailed different shapes across objects, and thus included some spatial attributes.

When templates are separated by space and time, template preparation has previously been associated with spatially lateralized contralateral alpha attenuation relative to the memorized location of the template (de Vries et al., 2017; van Driel et al., 2017). Our findings complement this recent work by isolating template identity, while controlling for spatial attention associated with the template. Moreover, as emphasized earlier, we here show this in a context in which the template was not presented to participants, but had to be retrieved from long-term memory based on a known probabilistic association between S1 and S2.

Snyder and Foxe (2010) demonstrated that when participants were cued to a relevant nonspatial feature dimension of a target stimulus (color or motion), alpha power was relatively attenuated in the area coding for the relevant feature dimension (dorsal visual stream regions for motion and ventral visual stream regions for color). This complements the idea that alpha attenuation may serve as a general attentional mechanism in perception. However, because this previous work cued feature dimensions (e.g., color) rather than feature values (e.g., red), it does not address whether alpha is also a relevant mechanism for expected identity or template preparation.

Interestingly, a previous study in which participants could prepare for a specific defining feature of a forthcoming target grating (Wildegger et al., 2017) found no evidence for modulations within the alpha band. The apparent discrepancy with the current finding could be due to statistical variability (i.e., a false negative in previous work) or reflect crucial task dependencies. For example, our task used complex stimuli, memory associations, and targets that were always presented centrally, whereas the previous work used simple orientations, symbolic cues, and uncertainty about target location.

In the current work, we focused on the process of template-guided attention. The instantiation of the target template putatively involves a process of retrieval from long-term memory, possibly followed by storage in visual working memory and accompanied by visual imagery. Retrieval from long-term memory storage (Hanslmayr et al., 2016; Staresina et al., 2016; Waldhauser et al., 2016; Fukuda and Woodman, 2017), prioritization of perceptual representations in working memory (Fukuda and Woodman, 2017; van Ede et al., 2017b; van Ede, 2018), and visual imagery (Slatter, 1960; Barrett and Ehrlichman, 1982; Salenius et al., 1995) have all previously been associated with attenuation of alpha oscillations. Our findings are thus in line with this large body of prior work. In contrast to this work, in the current study, these individual processes were never explicitly tasked to the participants. Rather, here, these processes may constitute the natural chain of events that support adaptive memory-guided perceptual anticipation.

### ERPs

In addition to the alpha effects, experiments 1 and 2 each also revealed significant ERPs associated with target identity anticipation. Moreover, like the alpha modulation, these potentials did not differ significantly between the predictive-easy and predictive-difficult S1s in experiment 1. The two ERP effects consisted of a CNV and a late posterior potential. Both of these have been found previously in associative learning tasks (Rose et al., 2001; Stokes et al., 2014). However, in this previous work, S1 predictions were coupled to response probabilities, a confound we ruled out in experiment 2.

The CNV is a classic signature of temporal and response anticipation (Walter et al., 1964; Donchin et al., 1975), and is likely to reflect the anticipation of the target, here shown to be strengthened by foreknowledge of the identity of the ensuing target.

Our late posterior positive potential may relate to the processing of S1 when it predicts a specific target or serves as a link between the S1 and the S2 item. The exact functional contribution of the late positive potential in our task is difficult to pinpoint. Its posterior topography and time course are compatible with a few different possibilities. Identification of the S1 as a relevant, predictive stimulus may have triggered a P300, which has a long history as a marker of stimulus relevance or meaning (Squires et al., 1975; Johnson, 1986; Polich, 2007). Alternatively, it may have reflected the process of recalling the associated target (Donaldson and Rugg, 1999), therefore providing a link between S1 and S2. A similar potential has also been noted during the orienting of spatial attention (Brignani et al., 2009), raising the possibility of an analogous mechanism for orienting attention to identity-defining stimulus attributes.

Importantly, in experiment 2, both the predictive and nonpredictive S1 indicated that a task-relevant target would appear in 1000 ms in the center of the screen, and all trials required a response. The only difference was that the predictive S1 indicates which item is likely to appear. Accordingly, this provides compelling evidence that these ERPs, like the alpha attenuation, are sensitive to the expectation of the particular identity of the forthcoming item.

It remains to be investigated whether the effects shown here are contingent on knowledge of the location and timing of an upcoming event. By design, space and time were always reliable in the current work. While contrasts with nonpredictive S1s allowed us to eliminate any neural correlates that were attributable to purely spatial and temporal predictions, we cannot rule out that the observed modulations might still reflect the interaction between identity-based anticipation and the known spatial and temporal attributes of the anticipated stimulus. That is to say, it is as yet unclear whether the same results would be obtained for identity-based predictions in the absence of spatial and temporal predictions. At the same time, of course, in the real world, spatial, temporal, and identity-based predictions are often bundled.

### Interaction between predictions and perceptual difficulty

In experiment 1, we found a significant interaction between S1 predictiveness and target difficulty (easy or difficult) for both error rates and reaction times. Interestingly, we did not find neural evidence for such an interaction in the identified alpha attenuation or ERPs during the period between S1 and S2. One may have expected that a more difficult target would call for a stronger activation of the perceptual template. However, our data do not speak to this conclusion. On the one hand, we cannot rule out differences in the extent of template preactivation that could not be detected with our methods. There may be other neural correlates of perceptual identity preparation that do depend on expected target difficulty, which we were unable to measure. On the other hand, the results invite us to consider whether and how similar levels of template activation may result in differential performance benefits. It is possible that the same perceptual templates will be more effective when incoming stimuli are harder to perceive. In this scenario, the consequences of preactivation of relevant neuronal populations may critically depend on the strength of neuronal activity triggered by incoming stimulation, playing a greater facilitatory role when incoming stimulation is weaker or more ambiguous.

### Conclusion

Together, our results suggest that proactive preparation for the identity of a target, based on successive associations impacts perception and is accompanied by the attenuation of alpha oscillations and modulations of ERPs, including the CNV. We here demonstrate this while matching spatial and temporal predictions, as well as target probability and response demands. While isolating identity anticipation has proven instrumental to our aims, we should also not forget that, in natural behavior, memory-based anticipation is often multifaceted, affording concurrent anticipation of the what, where, and when of upcoming percepts. In future studies, it will be interesting to consider systematically the dynamic interplay and potential synergies among each of these different dimensions of memory-based perceptual anticipation.
